# Assembly and Annotation of Escherichia coli Bacteriophage U115

**DOI:** 10.1128/mra.00949-21

**Published:** 2022-02-17

**Authors:** William An, Camilla Emsbo, Emma Frey, Vicky Hu, Ashley Jones, Nipa Latif, Makayla Perrilli, Krystalia Reillo, Joshua C. Schwarz, Sydney Strasner, Austin Theroux, Luz D. Vargas, Paul E. Turner, Alita R. Burmeister

**Affiliations:** a Department of Ecology and Evolutionary Biology, Yale University, New Haven, Connecticut, USA; b BEACON Center for the Study of Evolution in Action, East Lansing, Michigan, USA; c Program in Microbiology, Yale School of Medicine, New Haven, Connecticut, USA; Queens College CUNY

## Abstract

We present the annotated genome sequence of Escherichia coli bacteriophage U115, a T4-like bacteriophage. Phage U115 has a genome length of 166,986 bp and has 286 predicted genes.

## ANNOUNCEMENT

Previously described Escherichia coli phage U115 is of interest to the study of evolutionary trade-ups (1). The phage relies on the Tsx receptor, an outer membrane protein that also imports the antibiotic albicidin (1, 2). Consequently, an evolutionary trade-up can occur when phage resistance evolves through changes to Tsx that also block albicidin entry (1, 3). Phage U115 was isolated from wastewater influent in New Haven, CT, and characterized previously (1). A sample of phage U115 used in the current study was provided by Ben Chan (Yale University). We propagated the phage in Luria-Bertani broth at 37°C on E. coli K-12 strain BW25113.

DNA was isolated using a phage DNA isolation kit (Norgen Biotek). The sequencing library was prepared using the Illumina Nextera sequencing kit and sequenced on a Nextseq 2000 machine with a 300-cycle cartridge to give 150-bp reads. Genome assembly and annotation were conducted through the Center for Phage Technology (CPT) instances of Galaxy (4) and Web Apollo (5). Default parameters were used for all software unless otherwise specified. Sequences were rarified to a target coverage of 250× to improve assembly (6) using FASTQ Subset (7, 8). Sequence quality was assessed with FastQC v.0.72+galaxy1 (9). Low-quality sequence ends were trimmed with Trim Sequences v.1.0.2+galaxy0 (10) to reach a mean quality score above 30 across all bases and a 10th percentile quality score above 25 across all bases; this process involved trimming the first 18 bases and the last 1 base of each sequence. We also qualitatively confirmed that the per base sequence content of the trimmed sequences was consistent across the trimmed read lengths. The trimmed sequences were assembled using SPAdes v.3.12.0 (11) resulting in a circular contig of 166,986 bp and 119× coverage containing 55 bp of terminal overlapping sequence, which was removed manually before further analysis.

Using NCBI BLASTn (12), phage U115 was determined to be a tequatrovirus with a 94.63% identity and an 89% query coverage to phage T4 (GenBank accession number MT984581). The genome was reopened to be syntenic with phage T4.

The assembled sequence was run through the Galaxy phage annotation pipeline (PAP) structural workflow v.2021.02 (7) and imported into Apollo for structural annotation. Gene locations were predicted using GLIMMER3 v.0.2 (13), MetaGeneAnnotator v.1.0.0 (14), and Sixpack v.5.0.0+galaxy2 (15). tRNA gene calls were made with tRNAscan-SE v.2.0.5 (16) and ARAGORN v.0.6 (17). Finalized gene calls were confirmed by manual assessment of Shine-Dalgarno sequences, start and stop sequences, and distance between genes (18).

Functional annotation was initiated using the CPT PAP functional workflow v.2021.01 (7). Putative gene functions were assigned by manual assessment of BLASTp (19) results to the curated databases Canonical Phages, Swiss-Prot, and nonredundant (NR; NR phages only) along with InterProScan v.5.48-83.0 (20). Annotations were further verified separately using NCBI BLASTx (12) and InterPro (21).

Of the 286 predicted genes of phage U115, 144 were annotated as hypothetical genes, 131 were annotated with putative functions, and 11 were annotated as tRNA genes. EDGE bioinformatics (22) determined that the GC content of this genome was 35.42%, and there were no hits for virulence factors and deleterious genetic markers. PHACTS (23) predicted phage U115 to be “confidently lytic.”

This study uniquely represents a collaborative effort by 12 undergraduate researchers enrolled at 4 institutions, as follows: The University of New Haven (A.J., and K.R., and S.S.), Quinnipiac University (J.C.S., M.P., N.L., and E.F.), Southern Connecticut State University (L.D.V. and V.H.), and Yale University (A.T., W.A., and C.E.). All of the structural and functional annotations were completed during a 5-week, fully online summer research experience led by A.R.B.

### Data availability.

The annotated genome of phage U115 has been added to NCBI GenBank under accession number MZ753803. The BioProject accession number is PRJNA753771, and the Sequence Read Archive (SRA) number is SRR15420633.
